# Emerging computational paradigms to address the complex role of gut microbial metabolism in cardiovascular diseases

**DOI:** 10.3389/fcvm.2022.987104

**Published:** 2022-10-10

**Authors:** Javad Aminian-Dehkordi, Amin Valiei, Mohammad R. K. Mofrad

**Affiliations:** Department of Bioengineering and Mechanical Engineering, University of California, Berkeley, Berkeley, CA, United States

**Keywords:** cardiovascular diseases, microbiome, systems biology, genome-scale metabolic model, agent-based modeling, spatiotemporal dynamics

## Abstract

The human gut microbiota and its associated perturbations are implicated in a variety of cardiovascular diseases (CVDs). There is evidence that the structure and metabolic composition of the gut microbiome and some of its metabolites have mechanistic associations with several CVDs. Nevertheless, there is a need to unravel metabolic behavior and underlying mechanisms of microbiome-host interactions. This need is even more highlighted when considering that microbiome-secreted metabolites contributing to CVDs are the subject of intensive research to develop new prevention and therapeutic techniques. In addition to the application of high-throughput data used in microbiome-related studies, advanced computational tools enable us to integrate omics into different mathematical models, including constraint-based models, dynamic models, agent-based models, and machine learning tools, to build a holistic picture of metabolic pathological mechanisms. In this article, we aim to review and introduce state-of-the-art mathematical models and computational approaches addressing the link between the microbiome and CVDs.

## Introduction

Dwelling in the human gut is a complex microbial community made up of various cell types spanning a wide range of taxa (1). This diverse microbial habitat is demonstrated to strongly contribute to food metabolism, particularly the digestion of compounds that are hardly degradable by human cells, such as vitamins and amino acids (2–4), as well as non-digestible molecules such as complex carbohydrates (5, 6). It is widely believed that the metabolic function of microbiota plays a salient role in maintaining the integrity of intestinal mucosa (7), establishing a homeostatic state in the gut ecosystem (8), and preserving overall health. Besides food digestion, gut microbes are influential in drug metabolism by facilitating the biotransformation of exogenous compounds into biologically active products, thereby regulating host pathways for xenobiotic transport (9–13). Considering the broad role of the microbiome in health, deciphering microbial metabolic pathways is a subject of immense scientific interest.

The essential role of microbiota entails the digestion of food and drugs in the intestine before they enter the bloodstream and reach the target tissue (14–16), where a complex metabolic process involving the interaction between the host and the microbiota takes place (17). Although this association is symbiotic in nature, it is highly susceptible to perturbation by various environmental factors. Metabolites produced within the gut, for instance, vary according to changes in diet and nutrition which could, in some cases, adversely affect the host function. Microbially-produced short-chain fatty acids including acetate and butyrate, for example, can impair the metabolism of glucose (18), and the microbial endotoxin lipopolysaccharide can elevate the intestinal epithelial permeability, causing leaky gut (19). Changes in environmental factors could have more permanent effects when they modify the gut microbiome composition to become richer in detrimental phenotypes, resulting in an unbalanced microbiome composition (20, 21). An abnormal gut microbiome can undermine immunity and trigger a wide range of chronic immune-mediated disorders such as inflammatory bowel diseases depending on specific genetic characteristics of the host and the environment.

**Figure 1:**
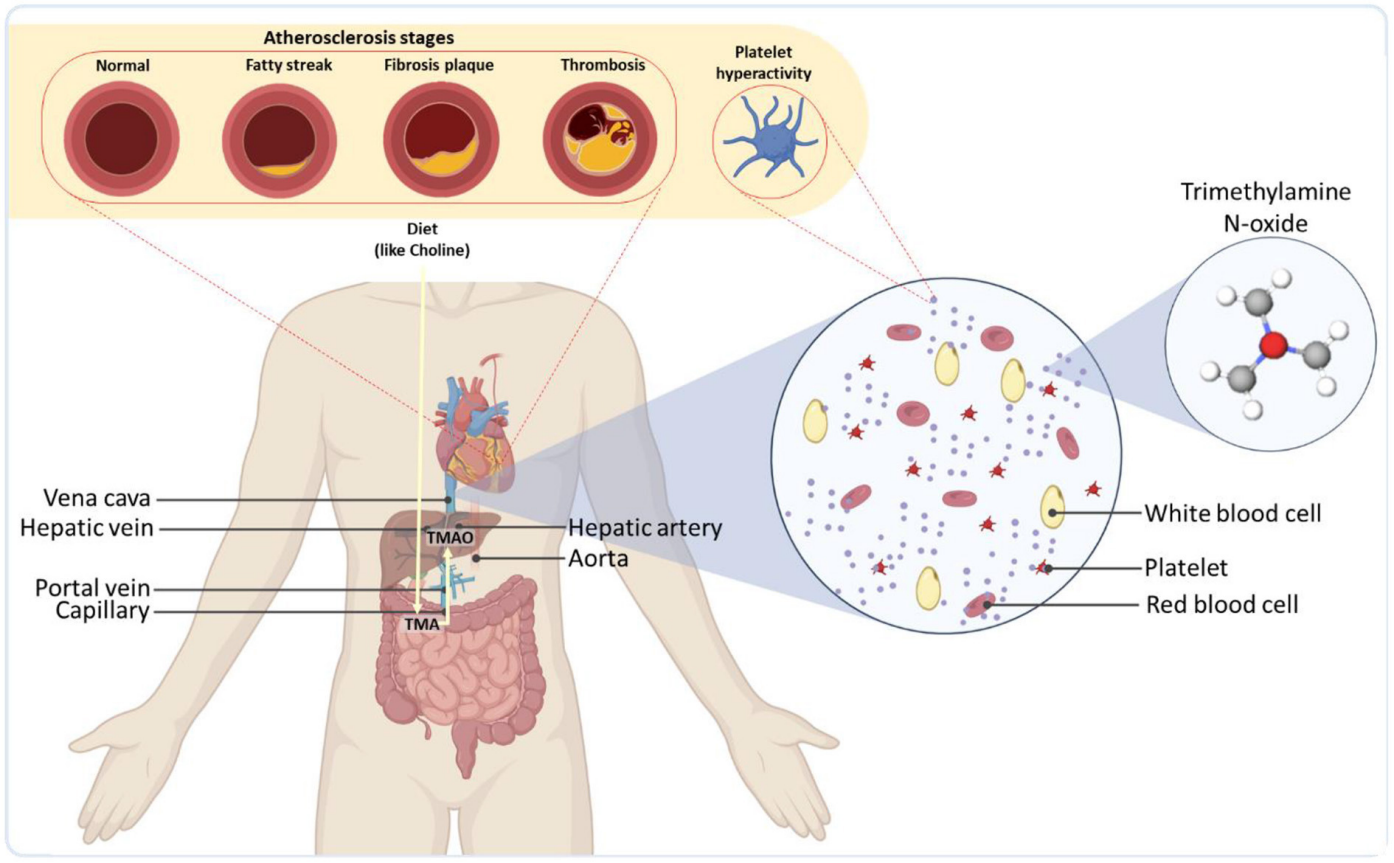
Overall representation of gut microbiota as a regulator of the cardiovascular systems of the body. As an example, TMA produced by the microbiome is transported to the liver through the portal vein and readily metabolized by host hepatic flavin monooxygenases into trimethylamine-N-oxide (TMAO). TMAO is then released into the bloodstream, leading to severe cardiovascular diseases.

The effect of incongruous gut microbiota is not usually limited to the gut but permeates beyond the gastrointestinal tract (GI) in the form of notorious diseases like liver fibrosis and cirrhosis (22). Once the food enters the bloodstream, it enters the liver through the portal vein, where undesirable metabolites interfere with normal hepatic functions. For example, dietary precursors, such as choline and carnitine, which are converted into trimethylamine (TMA) by the gut microbiota *via* specific genes (23), can be metabolized in the liver into Trimethylamine-N-oxide (TMAO) by host hepatic flavin monooxygenases (24) (see Figure 1). TMAO, in turn, has been reported to increase the severity of non-alcoholic fatty liver disease (25). Additionally, ethanol, a common product of microbial fermentation, is shown to increase enzymatic activity, leading to series of inflammatory reactions in the liver (26). Some compounds, such as short-chain fatty acids, can have opposing roles. While stearic acid induces inflammatory signaling (27), short-chain fatty acids have been reported to delay non-alcoholic fatty liver disease development and reduce blood pressure (28). Such trade-off correlations between metabolites make the identification of synergic effects of metabolites more complicated (29).

Even after hepatic digestion, numerous microbiome-derived compounds or the byproducts of their digestion circulate *via* the blood and spark serious disruptive effects in other organs. The cardiac system is a major site where these metabolites can give rise to severe health consequences by inducing cardiovascular diseases (CVDs) (30–32). TMAO has been realized to enhance plaque accumulation inside the arteries (atherosclerosis) (33), platelet reactivity and blood coagulation (thrombosis) potential (34, 35), blockage of arteries in coronary artery disease (36), the risk of heart failure, vascular inflammation (37, 38), as well as abdominal aortic aneurysm (39) (Figure 1). Some studies have shown that elevated levels of TMAO could also increase the accumulation of macrophage cholesterol and upregulate several macrophage scavenger receptors associated with atherosclerosis (40) [for more information on gut phage-bacteria interplay and the interactions of bacterial metabolites in cardiometabolic diseases, see (41)]. The microbiota could also modulate the metabolism of lipids and glucose by contributing to the synthesis of bile acids, the products of cholesterol via an interwoven metabolic network in the liver (42). The synthesis rate of bile acids, can be down-regulated by the gut microbiota, leading to an increase in the levels of low-density lipoprotein and eventually atherosclerosis (43).

Despite the significance of CVDs and their emerging etiological link to the microbiome (44), many questions on the microbiome-rooted pathologies remain to be addressed. Notably, the molecular mechanisms of microbe-microbe and microbe-host interactions must be elucidated to reveal dysregulation mechanisms mediated by the microbiome and discover new therapeutics (29). Conventional purely data-driven experimental approaches, such as measurement of metabolites in body fluids, are challenging to implement on a large scale, especially when expensive equipment with high selectivity is required to detect trace metabolites. These methods fail to provide a mechanistic picture of diseases. Furthermore, despite the rapid growth in microbiome research, the links between the microbiota and CVD pharmacology have remained underexplored. Systems pharmacology has garnered attention toward the detection of CVD risk factors by which drugs with amicable or adverse impacts are identified. Some antiretroviral therapy drugs could promote CVD through regulatory gene networks (45). While a framework focusing on gene-set enrichment analysis can be used to determine potential pathways with off-point drug effects, such approaches are often challenged by multiple variants affecting complex drug mechanisms in the microbiome (46). In situations where a great deal of influence is exerted by the microbiome on the human phenotypes, more robust investigations at the interface of microbiome and systems biology are called for.

Omics methods provide a more in-depth metabolic signature of diseases, yet they are inadequate to draw concrete mechanistic conclusions. Comprehensive systematic views, which involve systems-oriented techniques and computational modeling, complement experimentation to unveil the microbiome causality of CVDs. Herein, we aim to review the state-of-the-art computational approaches proposed to determine microbiome-CVDs interplay. Given the importance of data-driven omics approaches, we briefly review the recent advances in this topic, followed by an elaborate discussion of common approaches that can build on these technologies to model microbiome-based pathologies.

## High throughput data for characterization of the microbiome features

Advances in omics technologies have offered great insights into the biochemical processes interacting with the microbiome and CVDs by proving useful information on the regulatory role of different components, underpinning the equilibrium between human organs and the gut microbiota. Studies, for instance, have indicated that a variety of biological processes are regulated by microRNAs (miRNAs). miRNAs serve a key role in the host immunological response to counteract infections caused by bacterial pathogens (47, 48). Corroborating the role of miRNA in host-pathogen interplay, mounting evidence suggests that miRNAs can be overexpressed or under expressed by the microbiota in organs beyond the gut (49). Moreover, non-coding small RNAs (sRNAs) have been found to be important gene expression signals that regulate the microbiome. Allen et al. (50) reported that sRNAs trafficked by low-density lipoproteins can induce atherosclerosis. On the other hand, high-density lipoproteins that convey sRNAs, derived mostly from the microbial species within the gut, can act as biomarkers for atherosclerotic cardiovascular diseases (51).

Recent bioinformatics methodologies have boosted the capabilities of integrating data and identifying biomarkers and drug targets, presenting a new picture of treating gastrointestinal disorders including dietary interventions and their consequences on CVDs. The improvement of high-throughput technologies and culture-independent genomic methods over the past few years made it particularly possible to characterize the microbial ecology in great detail (52, 53). These approaches have fostered better diagnostic strategies by analyzing species abundance (54).

Microbiome composition and function have been determined with high-throughput omics technologies, such as metagenomics (55), metaproteomics (56), metabolomics (57), and metatranscriptomics (58, 59), which are currently obtained from colon biopsies, fecal samples, and colonic lavage [further discussion on the challenges in sample collection of clinical studies in the works of Kazemian et al. (60) and Ahmad et al. (61)]. There is abundant evidence of spatial heterogeneity in microbiota detected in the colon tissue and stool samples (62), resulting in the development of spatial multi-omics techniques, including deterministic barcoding in tissue for spatial omics sequencing (DBiT-seq) (63). DBiT-seq, derived from genome-wide expression measurements at high spatial resolution (64), is an organ-on-chip-based technique used to identify mRNA transcriptomes and protein markers.

To more specifically profile the bacterial composition, novel approaches like shotgun metagenomics sequencing have been employed. Metagenomics has revealed constructive information on the impacts of the microbiome on the health and disease status of hosts (31). Metatranscriptomics and metaproteomics are emerging as complementary methods of characterizing the active gene and protein repertoires within the gut microbiota. Analysis of fecal 16S data from different participants with CVDs indicated that a small number of informative bacterial taxa improves diagnostic classification and alleviates computational costs (65). On the other hand, metabolomic approaches have gained significant traction with advancement in mass spectrometry techniques. Metabolomics enables the study of host-dietary component interplay by providing a plethora of data from the desired organism, which serves as fingerprints of a physiological state (66). Besides, advanced machine learning tools and artificial neural networks assist researchers in the interpretation of genomics and raw metabolomics data. The application of a random forest-type machine learning classification method implemented on metagenomic sequencing of more than 1,200 bacteria from 1,098 individuals emphasized the association of microbial biomarkers of obesity with circulating blood metabolites as the indicators of cardiac diseases (67) [for more information see (68)].

## Constraint-based modeling of microbial crosstalk

Genome-scale metabolic models (GEMs; see Box 1 for a quick overview) offer a powerful tool to identify the genotype-phenotype associations in individual bacteria and microbial communities (70). GEMs are set up to describe cellular behaviors through multi-omics data combined with specific objective functions (71). To reconstruct GEMs, genome-wide sequences and similarity-based annotations are required. Although several automatic reconstruction and refinement tools have been developed (72), manual curation and the inclusion of specific experimental data remain the most critical steps during the reconstruction process. Given the complexity of the reconstruction process, it is usually more feasible to build GEMs progressively, starting from key gut bacterial strains and working up to more complex makeups. In this regard, using well-established gene databases and integrating them with multi-omics data can help to build high-quality metabolic models.

Box 1 **Constraint-based modeling.**

Metabolic networks based on genome annotations, and consequently enzymatic reaction, (i.e., they use genotype-phenotype associations), provide bases that can be used by stoichiometric methods. Constraint-based models (CBMs) have been shown as pragmatic tools to investigate genome-scale metabolic networks. CBMs in metabolic networks regularly come in a steady-state form with relevant constraints imposed on multiple reactions. This results in a bounded convex cone, which includes optimal solutions. Each point within this feasible solution space represents a single flux distribution, which contains fluxes of every reaction throughout the network. Flux balance analysis (FBA) is a common linear programming optimization problem that assumes metabolite accumulation is zero during the growth phase:

$$\begin{array}{l} \max/\min\sum_{j}c_{j}v_{j}\\ subject\sum_{j}S_{ij}v_{j}=0\\ v_{j}^{\min}\leq v_{j}\leq v_{j}^{\max} \end{array}$$

where $\underset{j}{\sum }c_{j}v_{j}$ is a linear function representing the cellular objective. The coefficients *c*_*j*_ determine the weights of the reaction *j*. Also, *v*_*j*_ and *S*_*ij*_ are the reaction *j*'s flux and the stoichiometric coefficient of metabolite *i* in reaction *j*. Also, *v*_*j*_ is constrained to $v_{j}^{\min}$ and $v_{j}^{\max}$. The constraint and direction of intracellular reactions are correlated with Gibbs free energy (G). Thermodynamically, a reaction with a negative ΔG can deliver non-zero fluxes [for more information, see (69)]. In most FBA studies, the biomass-producing reaction is set to be maximized as the objective function. Biomass-producing reactions represent the components of the desired cell including macromolecular content and associated metabolites expressed as a weighted ratio based on cell dry weight. Depending on the aim of a study, the objective function can be defined as the optimization of the desired metabolite produced by the cell.

Besides the automated and semi-automated approaches, machine learning techniques have evolved to improve GEM prediction by determining principal features from large-scale datasets. BoostGAPFILL, using a standard matrix factorization, obtains the metabolite adjacency matrix to predict possible candidate reactions from a reaction network (73). The results of this algorithm can also be used by FASTGAPFILL to weight reactions (74). AMMEDEUS has been proposed to identify the parts of a metabolic network that need to be improved using machine learning algorithms and ensemble analysis (75). To examine metabolic networks as a deep-learning-oriented approach, DeepEC has been developed, which can be adopted to predict enzyme commission (EC) numbers of protein sequences to precisely illustrate enzymatic functions (76). In addition to refining (75, 77), machine learning tools can also identify biomarkers to determine cellular phenotypes from different omics data (78–80). The mechanistic view of genome-scale networks integrated with machine learning tools is exceptionally useful for high-throughput data employed to design metabolic engineering experiments (81).

Several algorithms have been developed to model the principal metabolic crosstalk between microbes within the gut (see Box 2). A common approach is to develop a joint GEM for the whole microbial system using microbiome generation toolboxes (84, 85). This has been accelerated using semi-automated approaches like AGORA and AGORA2. AGORA is a validated assembly that includes 773 metabolic models of gut microorganisms (86). This GEM reconstruction tool has recently been improved to account for 7,206 strains using comparative genomics (87). The CoReCo pipeline (88), a reconstruction toolbox to model related species, was also used to reconstruct a refined GEM for *Candida albicans* with some improvement (89). The model was then paired with 910 gut bacteria GEMs to analyze the interactions and identify specific metabolites with inhibitory/activatory effects on the fungus.

Box 2 **Interactions between microbes.**

Species within the microbiome tend to interactively communicate and may exhibit significant temporal and spatial changes to the environmental signals. The underlying interactions are the main factors that contribute to the structure and function of the gut microbiota. Initial efforts were focused on developing a common GEM for a system with more than single species under steady-state conditions. Therefore, a combined biomass-producing reaction was used to be maximized. The idea of compartmentalized GEMs was then further developed. The initial idea was simple: one species adsorbs the metabolite produced by the other counterparts. Each GEM was assumed as a compartment with an additional tacit compartment to consider exchange reactions of GEMs. The key challenge focuses on how to coordinate metabolite exchanges between species. When it comes to the behavior of cells at the community level, single optimization is poor at predicting the nature of the community, while multi-objective optimization methods can maintain the association between individual-community levels properly and this was first introduced in OptCom (82). OptCom optimizes the biomass objective function of each individual as the inner objective and the community biomass objective function as the outer objective within shared metabolic networks.

$$\begin{array}{l} \max\;/\min v_{BOF}^{Community}\\ subject\;[\begin{array}{l} \max\;/\min\;v_{BOF}^{m}\\ subject\sum_{j}S_{ij}{}^{m}v_{j}{}^{m}=0\\ v_{j,\min}^{m}\leq v_{j}^{m}\leq v_{j,\max}^{m} \end{array}],m=1:\text{number of species}\\ \text{ Constraints for Community} \end{array}$$

Under steady-state conditions, the microbial community inhabiting the gut would behave regardless of gradients within the adjacent microenvironments. Like dFBA, d-OptCom was proposed to include the dynamics of the microbiome (83), where it integrates stationary flux distributions with kinetic models associated with substrate uptakes.

With the availability of robust GEMs for different strains, several constraint-based algorithms were implemented for broader inter-species modeling of microbial communities, like OptCom (82) and cFBA (90) to more recent ones like COMETS II (91) and IndiMesh (92) (see Box 2 for a brief overview of the algorithms). These algorithms feature the contribution of individual microorganisms to microbiome metabolism, host phenotype, and nutrient uptake. To generate the microbial communities, Basile et al. (93) used MMint (94) to simulate inter-species interactions. The simulations using a collection of 836 GEMs for anaerobic digestion microbiomes using genome-centric metagenomics suggested that exchanges related to amino acids have a germane role in solving auxotrophies. Furthermore, generating GEMs for bacteria from the predominant taxa identified in the human microbiome and subsequently performing flux balance analysis (FBA) to predict interactions, demonstrated how the gut microbiome and diet interact (95). MICOM, which is a customizable GEM, enables the integration of multiple GEMs, from a wealth of sequencing data available on different databases, with dietary data defined as the constraint (96). This framework provides a better understanding of alterations of the microbiome composition as it helps to quickly identify sets of individual growth rates and taxon-specific dilutions.

## Integration of multi-omics data into GEMs

**Figure 2:**
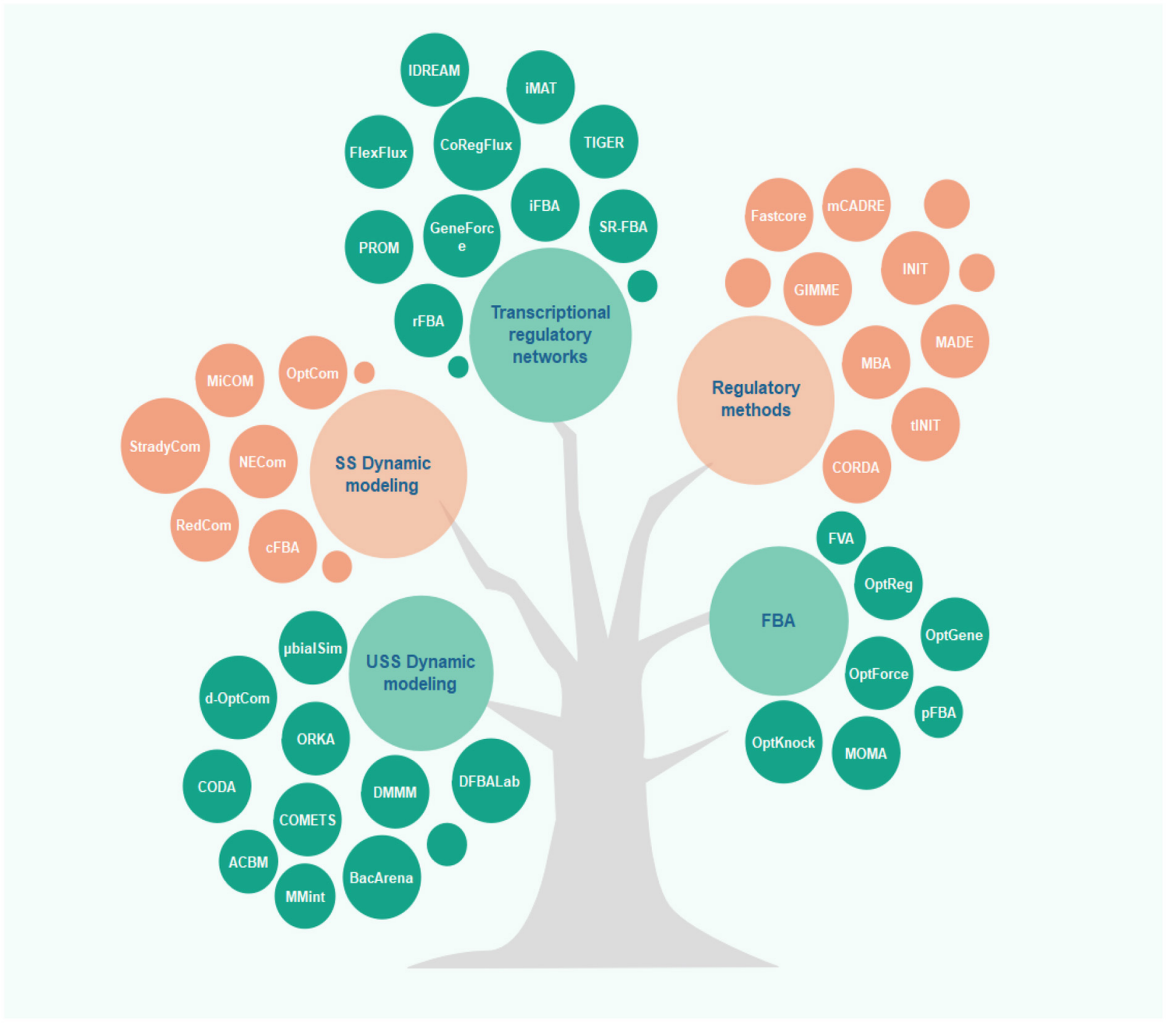
Overview of different algorithms used in microbiome-cardiovascular disease studies. Several constraint-based models have been proposed to predict and analyze the metabolic behavior of cells in recent years. This figure depicts these methods classified based on their applications and underlying algorithms (for more details, see the Supplementary Table).

Along with the development of constraint-based modeling of microbial communities, modeling of human metabolism has also drawn much attention. Rigorous context-specific GEMs can facilitate the development of new therapies and the prevention of metabolic diseases. The efforts for such human models began with the reconstruction of generic genome-scale network reconstructions (Recon) models and human metabolic reaction (HMR), recognized as the two most comprehensive generic GEMs for humans (97). Once combined with available high-throughput data, these generic GEMs can be rendered context-specific for different cell types. Integration of proteomics (98), metabolomics, and transcriptomics data is allowed by different algorithms to achieve cell-specific networks that provide a more accurate overview of cellular metabolism. In most algorithms, mixed-integer linear programming is applied. For this purpose, multiple algorithms are available, including MBA, iMAT, GIMME, INIT, FASTCORE, and mCADRE (for more details, see Figure 2 and the Supplementary Table). In all these algorithms, one type of omics -or more- and a GEM are given as the inputs to extract a tissue-specific model. While GIMME minimizes the usage of reactions encoded by low-expression genes, iMAT and INIT are free of low-expression genes using an optimal trade-off algorithm (99–101). In the MBA method, a group of active reactions based on high-expression genes is defined (102). FASTCORE and mCADRE are developed based on this assumption as well (103, 104). Also, CORDA as a non-minimalistic algorithm keeps down fluxes using cost-consuming reactions (105).

Despite the advancement of bio-informatic approaches for the integration of multiomics into metabolic models, there are still remarkable methodological shortcomings in the reconstruction of personalized GEMs, restricting their potential to grant reliable metabolic predictions and personalized treatment yield. In recent years, several context-specific GEMs, considering multiple individual data have been presented. For instance, Foguet et al. (106) performed personalized context-specific GEMs to illustrate the role of genotype variants on phenotypes subjected to prevalent human diseases, in a study on 524,615 individuals. Sometimes more than one type of omics is integrated with GEMs. The application of cross-sectional multi-omics data is not unprecedented in CVD-related studies. The integrative approach to examining the association between whole blood transcriptomics and fasting serum metabolomics emphasized the significance of multi-omics in the search for disease mechanisms (107). Aiming to link the plasma lipidome to CVDs, large-scale genome-wide analyses of different lipid species accompanied by several CVD-related phenotypes alluded to the functions of lipid loci, including *LPL* and *FADS2* on CVDs (108). This investigation successfully illustrated the merit of genetic regulation of lipid metabolism for discovering biomarkers for preventative purposes. The integration of genotype data from the METabolic Syndrome In Men (METSIM) cohort and NMR metabolic profiling suggested the need for more comprehensive computational approaches when it comes to high-throughput data (109).

The ambiguities associated with CVD mechanisms need to be uncovered using GEMs and other pragmatic strategies. For instance, glucose 6-phosphate potential in activating mammalian target of rapamycin and regulating glycolytic flux was investigated using a kinetic-type model for cardiomyocyte, *CardioGlyco*. The results revealed that phosphoglucose isomerase activity is a function of glucose 6-phosphate and it directly regulates the mammalian target of rapamycin and, consequently, myocyte growth (110). In another case study, a cardiomyocyte-specific GEM, *iCardio*, was reconstructed to explore whether the metabolic profile of all heart failure is similar (111). Arif et al. (112) used transcriptomic data to reconstruct a cell-specific GEM and investigated metabolic alterations that emerged after myocardial infarction. They reported a set of gene clusters associated with myocardial infarction in the heart and liver. A new reaction-centric method, TIDE (tasks inferred from differential expression), was proposed to identify metabolic function variations in cardiac failure gene expression. Using high-throughput RNA-seq data, thanks to a cell-specific GEM, the deregulation of metabolic behavior prompted after myocardial infection in the heart was identified. This study demonstrated the utility of such integrated multi-tissue analyses to systematically unravel the underlying metabolic roots of diseases. For a brief phenomenological overview of the integration of omics with a GEM, see Box 3.

Box 3 **Integration of omics with a GEM.**

Metabolic modeling of human tissues is essentially more challenging than that of prokaryotes since each tissue has different metabolic behaviors and functions. To overcome these hurdles, researchers tried to formulate a mixed-integer linear programming problem and link GEMs to gene expression networks, protein expressions, etc. of a given tissue by applying multiple algorithms. The underlying concept is straightforward: narrowing down the feasible solution space. To link thermodynamic constraints into GEMs correlated with omics data, synchronizing thermodynamically GEM methods, such as Relative Expression and Metabolite Integration (REMI) (113), have been developed, reducing the number of alternative optimal solutions within the feasible space.

**Figure 3:**
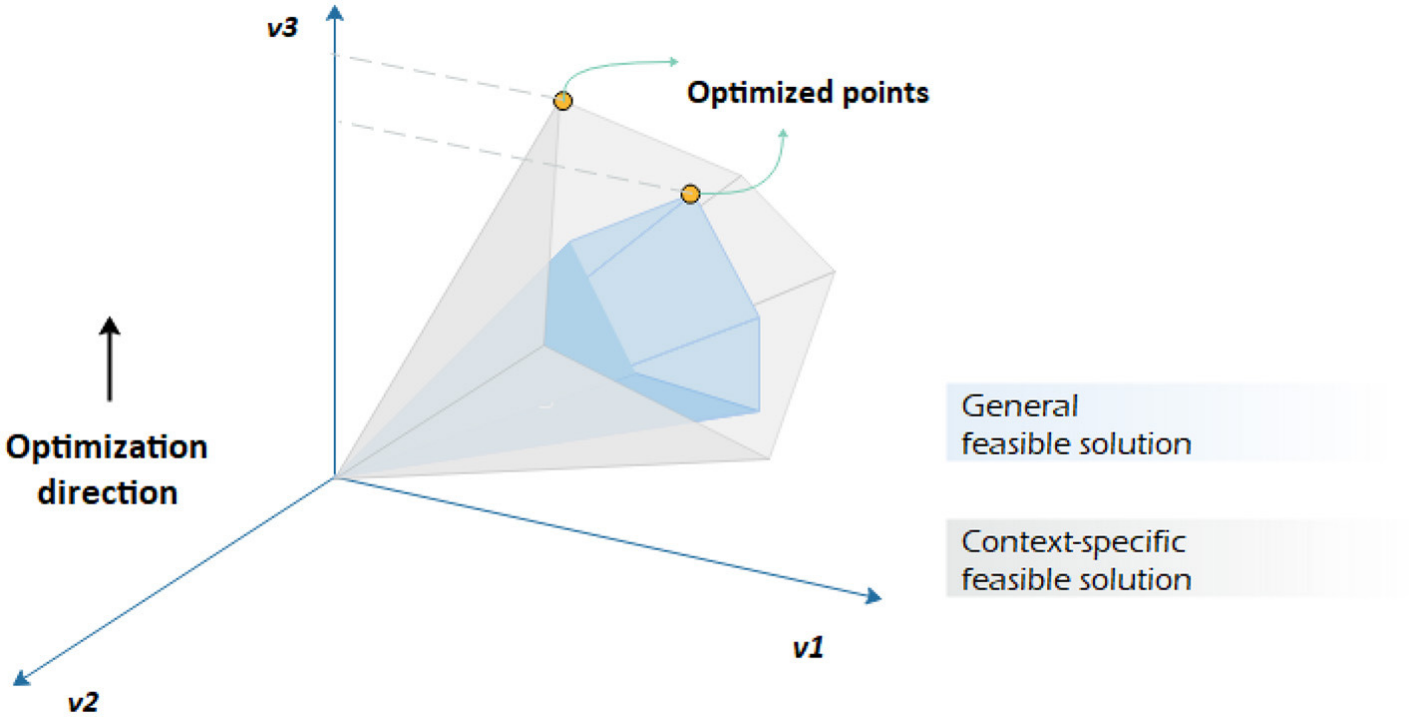
Constraint-based analysis by imposing different constraints on the same genome-scale metabolic network. It is assumed that *v*_3_ is to be maximized. Having a context-specific genome-scale metabolic model leads to a different convex-cone feasible solution with a different flux distribution.

The sole application of one type of data, e.g., transcriptomic data, might not lead to explicit results. The integration of multi-omics data is necessary to better understand how phenotypic behavior of metabolic pathways changes in different environmental and even genetic conditions. The variation can be captured by machine learning tools like the support vector method and used as references to regulate metabolic model constraints for a narrower feasible solution space (see Figure 3).

Considering disturbances existing within high-throughput data, machine learning approaches have boosted the quality of the integration process. To distinguish hidden features of metabolomic data, FIngerID (114) and BioTransformer (115) identify metabolites from mass spectroscopy datasets. Some data-driven approaches have recently been used to integrate fluxomics and transcriptomics data as well (116–118). Moreover, algorithms based on kernel matrices for heterogeneous high-throughput data have been developed which provide a platform to investigate the metabolic interactions of microbiome-CVDs (65, 119). Recently, the application of machine learning for identifying dysbiosis of gut microbiota has been highlighted, which makes the diagnostic screening of CVDs feasible.

## Frameworks to describe the dynamics and spatial insights of gut microbiome

Human gut microbiota exhibits a great deal of spatial and temporal heterogeneity. Microbial populations in the intestine significantly vary in both the cross-section and length of the gut. Spatiotemporal features play a fundamental role in a comprehensive understanding of the human-microbiome interaction since the microbiome fluctuates based on transient environmental factors. Since a bacterial imbalance at one position in the gut could precipitate a local inflammatory response (120), developing microbiome-focused therapies requires quantitative predictions of these dynamics.

Different modeling approaches have been used so far to predict the dynamical behavior of microbial communities. Formerly, ordinary differential equations (ODEs) drew attention for simulating the behavior of consortiums where time was considered as the independent variable. Initial dynamic models applied a quasi-steady-state (121) assumption for intracellular balances, while they assumed the extracellular functions to be changed at each regular time step. The dynamic flux balance analysis (dFBA) method uses GEMs integrated with some kinetic equations (122). This approach leads to a set of ODEs integrated with linear programming equations with an ODE solver (123). Due to the complexity of constraint-based modeling of humans, these rudimentary dynamic flux balance analyses are uncommon, particularly when spatial variations are of interest. The dFBA method can be further applied to account for spatial effects by discretizing the computational domain as well. Population-based approaches were among the initial strategies to garner attention. After Biggs and Papin predicted the biofilm formation of *Pseudomonas aeruginosa* (124), Bauer et al. (125) presented BacArena to analyze multispecies communities. They predicted the phenotype of seven species as representatives of the human gut microbiota as well as the competition for nutrients generated due to gradients. Then, multi-level optimization approaches were developed to represent the composition of the community over time using multi-objective functions. In addition to OptCom (82) and d-OptCom (83), μbialSim (126), which assumed a well-mixed homogeneous culture condition, was developed featuring multi-level optimizations. These systems-oriented optimization methods were successfully applied to the gut microbiome of humans and predicted the main metabolites. These approaches can potentially be adapted to optimize host metabolic behavior by designing appropriate diets. Integrative analyses with the aim to identify diagnostic biomarkers and propose new therapeutic interventions are also allowed in the presence of transcriptomics data.

## Multiscale modeling of the gut-cardiovascular axis

**Figure 4:**
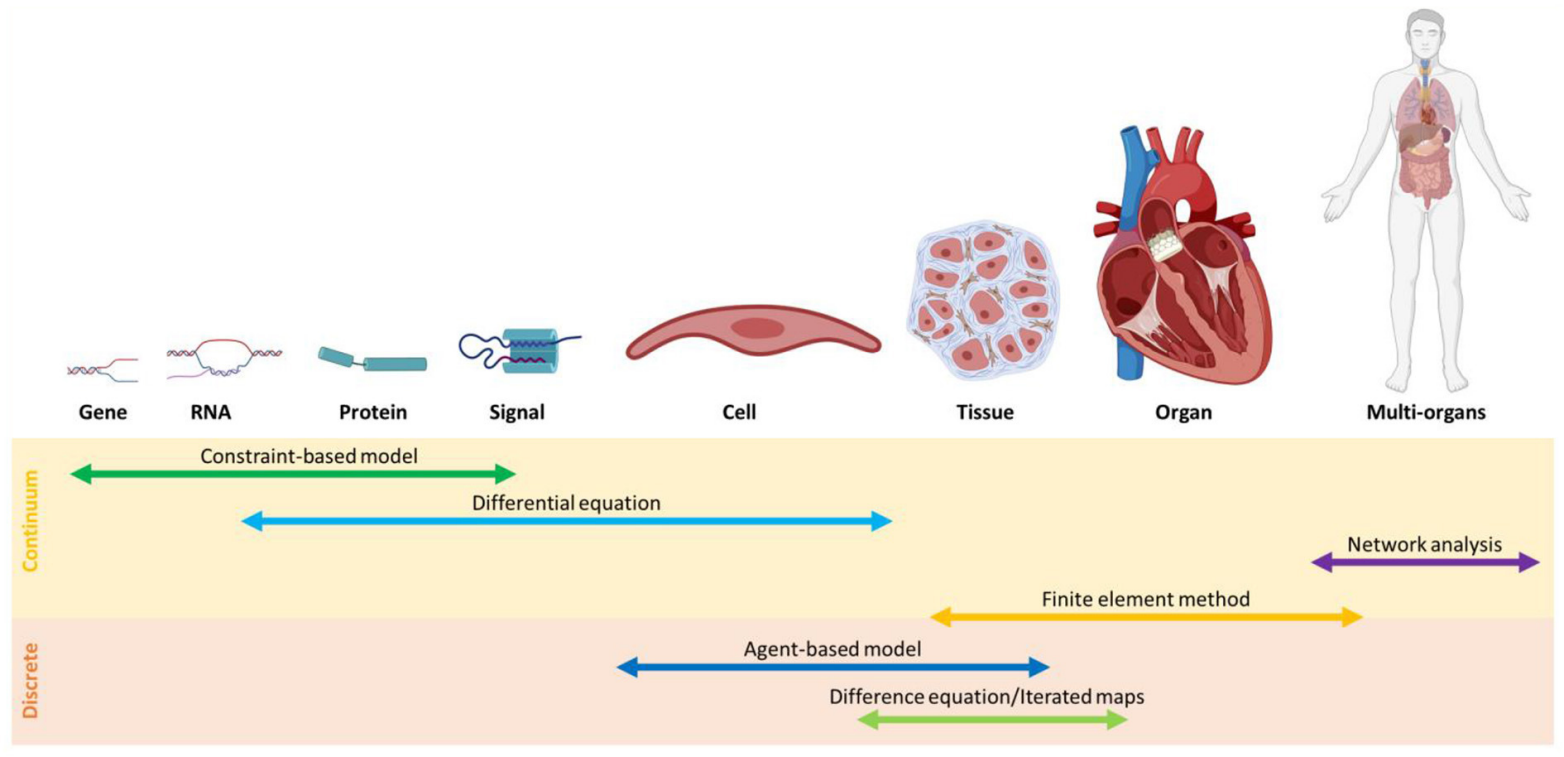
An overview of modeling methods at different scales classified, into continuum and discrete models.

The main challenge in the investigation of the gut-heart axis is to identify the interactions between the individual gut species and CVD progression, important pieces of information that can introduce potential new paths for drug discovery. Since it is unfeasible to investigate all microbiome dynamics experimentally, multiscale models are promising approaches. To this end, the selection of appropriate multiscale models is crucial (see Box 4). To predict microbiome-CVDs interplay, we need to bridge data between cell/tissue scales to genetic scales. Information from a single cell is inadequate, and at times, a fundamental correlation at the community level is necessary. To address this issue, the application of suitable discrete models such as agent-based modeling and continuous models such as constraint-based modeling is essential. In comparison, discrete models are useful when the roles of individuals need to be determined; continuous models, on the other hand, are more computationally cost-effective (128) (Figure 4).

Box 4 **Multiscale models.**

**Figure 5:**
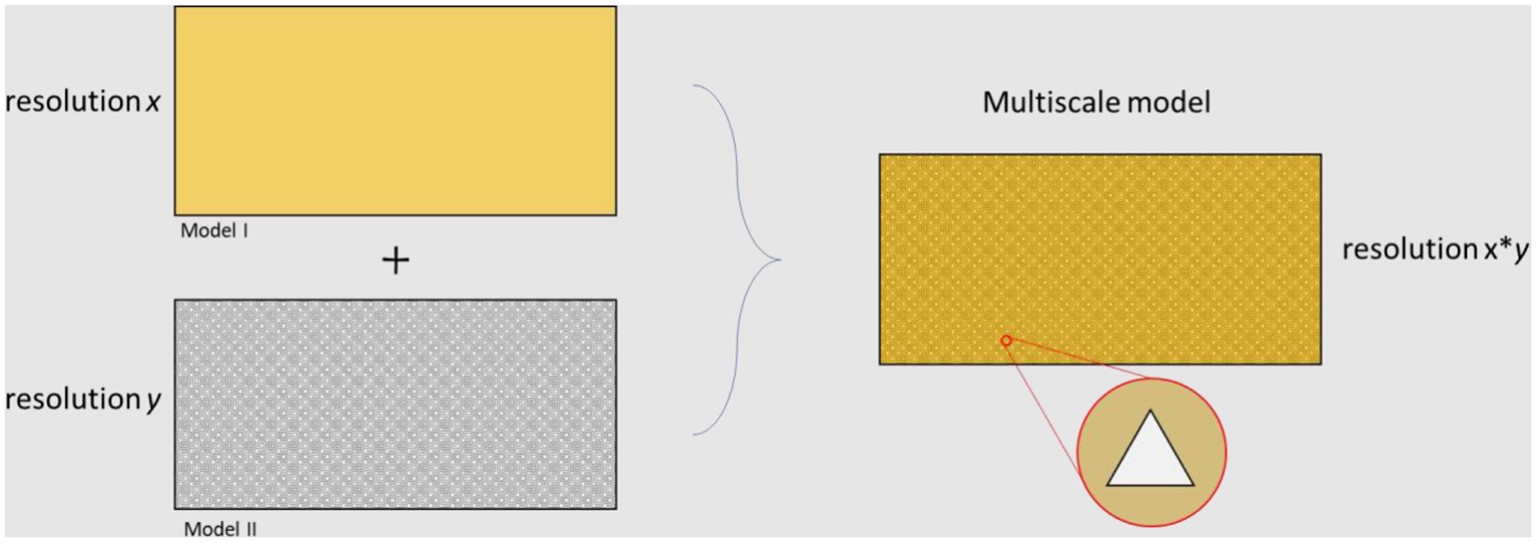
Overview of different levels of resolution related to different modeling approaches. The final multiscale model is able to capture results at both resolution *x* and resolution *y*.

The microbiome is a complex network interacting between different temporal and spatial scales. Multiscale models are used when answering a scientific question needs information from different resolutions. There are gaps between different measurable scales and multiscale models are intended to bridge them. Figure 5 represents the differences between the types of results achieved by the application of models at different levels. There are several approaches for developing a multiscale model. In some cases, submodels should be solved in parallel, while it is common to simulate independent approaches and apply the outputs as inputs for further resolutions. In terms of multiscale modeling of cardiovascular diseases, several top-down and bottom-up methods have been generated [for more information see (127)].

Sometimes more fundamental knowledge about the system interactions is required than what is provided in the previous section. For such complex systems in which the interactions between “individuals” have not been formulated into mathematical relations, agent-based models (ABMs) prove to be particularly useful. This framework allows researchers to encode the intricacies of a multi-bacterial system into a series of relatively simple rules that can be tuned more precisely to determine the underlying functions (129). In ABMs, microbes are defined as decision-making agents interacting with each other and their environment (130). Decisions are set based on heuristic rules depending on the objective of the study. Several agent-based models, including Stochsim (131), AgentCell (132), Smoldyn (133), etc., have been proposed to investigate interactions, either extracellular or intracellular, with different details. GutLogo, based on the NetLogo (134) agent-based modeling framework, is a new tool to model operating parameters and dynamics of gut microbiota (135). This method was developed to analyze the interactions that emerged by populations of *Clostridium, Desulfovibrio*, and *Bifidobacterium* as well as their metabolic functions. In terms of availability, MESA and AgentPy are recent open-source Python frameworks for implementing and analyzing ABMs and can be integrated with a wide range of Python libraries (136, 137).

The application of ABMs to cardiac fibrosis or platelet aggregation has been previously studied (138, 139). Cardiac fibrosis is an important part of cardiac remodeling that leads to heart failure and death, while platelet aggregation is part of the sequence of events leading to the formation of a clot. Such modeling approaches, mostly done at multiple scales, can make predictions at cellular and tissue levels that are merely possible *via* logic-based models (139).

## Future directions

Using computational approaches highlighting biochemical processes involved in CVDs, different types of genotypic and phenotypic information are encoded into mathematical models. The tool can be used to mitigate CVDs at different stages, simulate cellular growth under different environmental conditions, and give hypotheses to be investigated by *in-vitro* and *in-vivo* platforms. GEMs integrated with other computational models at different scales can be used to investigate the role of individual species at the community level and their metabolic interactions with cardiometabolic diseases. Interventions hampered by experimental limitations can be identified to define clinical scenarios and elucidate underlying disease mechanisms.

Although the widespread use of metabolic modeling is promising, several shortcomings limit their applications in that they are limited to biochemical interactions, while the roles of signaling and gene regulatory components are disregarded. It is also crucial to find new methodologies, based on personalized prediction, to alleviate uncertainties that emerge as the result of mapping GPR associations. Future efforts should be emphasized on establishing universal GEM reconstruction protocols with the aim of minimizing uncertainties arising in metabolic modeling of microbiome-CVDs crosstalk.

## Conclusion

In the healthcare industry, it is essential to find ways to provide personalized medicine, which maximizes the efficiency of treatments and reduces side effects while keeping costs down (140). The ultimate goal of precision medicine is to identify risk factors per individual and maximize personalized treatment benefits, which is different from current population-based therapies (141). Specifically, preventative and therapeutic practices based on population interventions are practical for only a specific portion of the community. Therefore, while this perspective has opened new avenues to defining novel treatment strategies, it presents new challenges in working with new data generated.

To enable personalized prevention, diagnosis, and treatment of diseases such as CVDs new systems-oriented approaches have been used to study the structural characteristics of the microbiome to elucidate the causal mechanisms. Given the importance of the gut microbiome in human health, researchers have performed different types of studies to further reveal the behavior and structure of the gut microbial communities as well as their unknown interactions with the host. Advancements in high-throughput data equipment make the availability of different omics easier. However, even with access to this wealth of information, predicting the behavior of microbiome-host interplay is often still burdensome. Accordingly, mathematical tools and computational approaches have been deployed to better grasp these heterologous data.

The emergence and integration of different meta-omics data, e.g., integration of time-series data, has made it more sensible how microbial communities interact with the human host and respond to disturbance. On the other hand, GEMs, particularly when integrated with omics, provide us with a great understanding of underlying mechanisms associated with CVDs. Several regulatory methods to define more accurate constraints and bi-level optimization algorithms to model the growth and interactions of the gut microbiota have been proposed; some of these can also be applied to study microbiome-host interactions. Combining the essential assumptions and theories from different types of models yields even deeper insight into communities. Mounting evidence from microbiome studies confirms the possible role of metabolites produced by bacterial communities in treating cardiometabolic diseases. In order to prevent CVDs and prevent early intervention, microbiome and host-diet interplay can promote personalized nutrition.

The future focus should be on systems-oriented studies of microbial metabolites to modulate host physiology more effectively. Systems biology-type studies should be conducted *via* a multidisciplinary perspective featuring the collaboration between researchers with clinical and engineering backgrounds to render effective personalized treatment.

## Author contributions

All authors listed have made a substantial, direct, and intellectual contribution to the work and approved it for publication.

## Conflict of interest

The authors declare that the research was conducted in the absence of any commercial or financial relationships that could be construed as a potential conflict of interest.

## Publisher's note

All claims expressed in this article are solely those of the authors and do not necessarily represent those of their affiliated organizations, or those of the publisher, the editors and the reviewers. Any product that may be evaluated in this article, or claim that may be made by its manufacturer, is not guaranteed or endorsed by the publisher.
