# Supplementary material for: Emerging computational paradigms to address the complex role of gut microbial metabolism in cardiovascular diseases
Source: Front Cardiovasc Med. 2022 Oct 10;9:987104. doi: 10.3389/fcvm.2022.987104 (PMC9589059; doi:10.3389/fcvm.2022.987104)
Supplement: Supplementary file 1 [file Table_1.DOCX]

Table- Different algorithms used in microbiome-cardiovascular disease studies classified based on their applications

| **Category** | **Algorithm** | **Description** |
| --- | --- | --- |
| FBA | FVA | Analyzes how the feasible ranges of stationary fluxes in a metabolic network would change when switching from the wild- type to a desired phenotype |
|  | OptReg | Allows for up- and/or downregulation in addition to gene knockouts to meet a bioproduction goal |
|  | OptForce | Make use of flux measurements available for the wild-type strain |
|  | Optknock | Suggest reaction deletion strategies that maximize bio- chemical production |
|  | OptGene | Optimizes the set of gene knock- outs that maximize a given objective function |
|  | pFBA | Computes a flux distribution that minimizes the sum of all fluxes in the model while satisfying a particular objective |
|  | MOMA | Examine the growth characteristics of mutant strains, in which specific genes have been deleted |
| Regulatory methods | Fastcore | Defines an active core reaction set that is guaranteed in order to find the minimum number of reactions possible to support the core |
|  | mCADRE | Defines a set of core reactions and prune all other reactions based on their expression, connectivity to core and confidence score |
|  | GIMME | Minimize usage of low-expression reactions while keeping the objective |
|  | MBA | Defines high-confidence reactions to ensure activity in the extracted model |
|  | INIT | Finds the optimal trade-off between including and removing reactions based on their given weights |
|  | MADE | Based on expression data of various conditions, binarily approaching to a metabolic network for expression dynamics |
|  | tINIT | Processing INIT algorithm, satisfying metabolic tasks, which is defined by users |
|  | CORDA | Minimizes additional cost, which is higher in undesirable reactions |
| Transcriptional regulatory network | iFBA | Combine FBA with regulatory Boolean logic, and ordinary differential equations |
|  | SR-FBA | Identifies a metabolic–regulatory steady state for the integrated metabolic–regulatory model |
|  | TIGER | Integrates transcriptional regulatory networks, genome-scale metabolic models, and transcriptomics |
|  | iMAT | Finds the optimal trade-off between including high-expression reactions and removing low-expression reactions |
|  | IDREAM | Develops integrated regulatory-metabolic models, which are then optimized for target product synthesis |
|  | CoRegFlux | Predicts fluxes with reverse-engineered transcriptional regulatory networks |
|  | GeneForce | Integrates transcriptional regulatory networks and genome-scale metabolic model in the SBML format |
|  | FlexFlux | Integrates transcriptional regulatory networks, genome-scale metabolic models, and transcriptomics |
|  | PROPM | Uses transcriptomics and TF–target relationships to integrate an expanded continuous transcriptional regulatory network |
|  | rFBA | Simulates growth under different environmental and genetic perturbations |
| Steady state dynamic modeling | OptCom | Predicts inter-species metabolite transfers |
|  | MICOM | Infers Metabolic Interactions in the Gut Microbiota |
|  | NECom | Maximizes each taxa’s biomass flux as inner level and community biomass flux as the outer |
|  | StradyCom | Predict composition (species abundances) of microbial community in a given environment |
|  | RedCom | Predicts feasible ranges for metabolite exchange rates and product yields |
|  | cFBA | Maximize the community’s specific growth |
| Unsteady state dynamic modeling | µbialSim | Simulation of microbiomes, where metabolite exchange is the primary means of interaction |
|  | d-OptCom | Maximizes community biomass concentration as the community- level objective |
|  | ORKA | Offers improved numerical stability by replacing the Euler forward method with a Runge-Kutta method |
|  | COMETS | Outputs can be from all or selected time steps. Predicts biomass spatial distribution for each simulation grid cell |
|  | ACBM | Models spatiotemporal dynamics at single-cell scale |
|  | MMint | Reconstructs metabolic models and predicts growth rate |
|  | DMMM | A system of ordinary differential equations (odes) representing the conservation of mass for the shared metabolites and cell biomass for microbial taxa |
|  | BacArena | Predicts cross-feeding interactions Metabolic turn over using metabolite concentrations as constraints |
|  | DFBALab | Addresses both infeasible FBA problems and degeneracy in production/consumption fluxes of shared metabolites |
